# Potential Antiviral Compounds from *Hippeastrum puniceum* Bulb Against Yellow Fever Virus: Bioassay-Guided Fractionation and *In Silico* Pharmacokinetic Analysis

**DOI:** 10.3390/molecules30214149

**Published:** 2025-10-22

**Authors:** Eliza Flores-Souza, Alisson Samuel Portes Caldeira, Carolina Colombelli Pacca-Mazaro, Tamiris Vanessa Miguel de Souza, Thaís Magalhães Acácio, Emerson de Castro Barbosa, Naiara Clemente Tavares, Carlos Eduardo Calzavara-Silva, Carlos Leomar Zani, Douglas Eduardo Valente Pires, Tânia Maria de Almeida Alves, Jaquelline Germano de Oliveira

**Affiliations:** 1 Grupo de Pesquisa em Imunologia Celular e Molecular, Instituto René Rachou-Fiocruz Minas, Belo Horizonte 30190-002, MG, Brazil; efsouza@aluno.fiocruz.br (E.F.-S.); carolina.mazaro@fiocruz.br (C.C.P.-M.); tsouza@aluno.fiocruz.br (T.V.M.d.S.); emersoncb7@gmail.com (E.d.C.B.); naiara.paula@fiocruz.br (N.C.T.); carlos.calzavara@fiocruz.br (C.E.C.-S.); 2 Grupo de Pesquisa de Química de Produtos Naturais Bioativos, Instituto René Rachou-Fiocruz Minas, Belo Horizonte 30190-002, MG, Brazil; alisson.caldeira@fiocruz.br (A.S.P.C.); thais.galhes@gmail.com (T.M.A.); carlos.zani@fiocruz.br (C.L.Z.); tania.alves@fiocruz.br (T.M.d.A.A.); 3 School of Computing and Information Systems, The University of Melbourne, Melbourne 3010, Australia; douglas.pires@unimelb.edu.au

**Keywords:** antivirals, wild-type yellow fever virus, *Hippeastrum puniceum*, secondary metabolites, bioassay-guided fractionation, In silico ADMET prediction

## Abstract

Despite the availability of effective vaccines, yellow fever outbreaks persist, highlighting the need for antiviral drugs. Background/Objectives: This study investigated *Hippeastrum puniceum* (Amaryllidaceae) as a potential source of antiviral compounds against wild-type yellow fever virus (wt-YFV). Methods/Results: The crude bulb extract of *H. puniceum* exhibited 58% protection against wt-YFV. Bioassay-guided fractionation of the extract by UHPLC-HRMS led to the annotation of six alkaloids (bulbisine, cathinone, trigonelline, tetrahydroharman-3-carboxylic acid, and 2,7-dimethoxyhomolycorine or 3-O-acetylnarcissidine) in active fractions, along with the amino acids arginine, asparagine, tryptophan, and glutamic acid. *In silico* ADMET analyses predicted favorable pharmacokinetic and toxicological profiles, supporting their potential as drug candidates. Six of the annotated compounds were evaluated *in vitro* for cytotoxicity and antiviral activity against wt-YFV. However, none showed significant antiviral activity when tested individually, suggesting that the observed antiviral effect may result from synergistic interactions between two or more compounds within active fractions. Conclusions: Our results underscore the importance of further investigations *in vitro*, particularly assays exploring the synergy among the annotated compounds against YFV. The integration of bioassay-guided fractionation of active plant extracts with computational analyses emerges as a promising strategy for the discovery of natural products with therapeutic potential against yellow fever, a reemerging disease.

## 1. Introduction

Yellow fever (YF) is an acute febrile illness of short duration and variable severity caused by the yellow fever virus (YFV), one of the most dangerous emerging/reemerging flaviviruses still circulating in several countries [1]. The YFV is transmitted to humans through the bite of infected *Aedes aegypti* mosquitoes, responsible for its urban transmission cycle. Moreover, *Haemagogus* and *Sabethes* mosquitoes are responsible for its sylvatic cycle [2].

It is worth mentioning that the YFV was renamed by the International Committee on Taxonomy of Viruses (ICTV) in 2023 as *Orthoflavivirus flavi* [3,4]. Between July 2016 and July 2018, an outbreak of yellow hemorrhagic fever was reported in Brazil, resulting in 2155 human cases and 745 deaths [5,6]. Currently, 47 countries, including Brazil, are considered endemic for the disease [7]. It is estimated that the actual number of yellow fever cases is 10 to 250 times greater than the number of reported cases, due to underreporting. This underreporting may be due to asymptomatic infections or mild infections with nonspecific symptoms [7,8].

Despite the availability of an effective and cost-free yellow fever vaccine in Brazil since 1939, some areas still have low distribution and vaccination coverage, which favors yellow fever outbreaks [9]. In addition, a considerable percentage of individuals cannot be vaccinated against yellow fever, including those allergic to egg proteins present in the 17DD vaccine formulation used in Brazil, and immunosuppressed patients [10,11].

Approximately 15% of individuals with symptomatic yellow fever develop the toxic form of the disease, known as “hepatorenal hemorrhagic syndrome,” which is associated with a high mortality rate ranging from 40% to 70%. Although there are studies on therapeutic targets against YFV, such as the Tripartite Motif Containing 56 (TRIM56) protein, which inhibits the initial replication of the virus through its E3 ubiquitin ligase activity, and the Transmembrane Protein 41B (TMEM41B), which interacts with viral proteins like NS4B, no approved medications are available for the specific treatment of yellow fever [11,12]. Given the lack of specific treatment for yellow fever and the high mortality rate in the toxic phase, there is an urgent need to develop antiviral drugs to reduce the viral load in patients [10,13].

Medicinal plants are promising sources for discovering and developing sustainable, efficient, and cost-effective alternatives against viral infections. Crude plant extracts may contain a variety of secondary metabolites with bioactivity. Therefore, bioassay-guided fractionation of these extracts has proven to be an advantageous strategy for identifying bioactive compounds, aiming at developing new drugs, including antivirals [14].

The Amaryllidaceae family has garnered significant attention for its alkaloids, which are notable for their valuable pharmacological properties [15]. More than 600 structurally different alkaloids have been isolated from plants in this family. Although exhibiting considerable structural diversity, Amaryllidaceae alkaloids originate from a common precursor, norbelladine. Alkaloids are responsible for a wide range of biological activities, including antiparasitic, antitumor, anticholinesterase, antifungal, and antiviral activity [16,17,18]. A recent literature review study conducted by Nair and Van Staden showed that isoquinoline alkaloids from plants of the Amaryllidaceae family exhibited potent in vitro activity against YFV, possibly through mechanisms of inhibition of viral enzymes (neuraminidase, protease and reverse transcriptase), inhibition of nucleic acids, as well as blockage of protein synthesis [19]. Bulbs of some species belonging to this family are used in traditional medicine such as emetics, purgatives, wound healers, and remedies for stomach aches [20,21].

Plants of the genus *Hippeastrum* are widely distributed in South America and occur in Brazil in areas of sandbanks and Atlantic Forest [22]. This genus has been considered a promising source of bioactive compounds for scientific and industrial exploitation [17]. The *Hippeastrum puniceum* exhibits a complex phytochemical profile, featuring numerous alkaloids (including lycorine, tazettine, and 9-O-demethyllycoramine) [23], as well as flavonoids (quercetin, kaempferol), saponins, tannins, and terpenoids [24]. The diversity of these metabolites, combined with their potential synergistic interactions, may account for the wide range of biological effects observed for *H. puniceum*, reinforcing its pharmacological potential.

Bulb extracts of *H. puniceum* were recently chemically characterized by gas chromatography-mass spectrometry (GC-MS), and fifteen alkaloids were identified from an ethyl acetate bulb extract. Lycorine was the major compound, and the alkaloids pseudolycorine, 9-O-dimethyllicoramine, and pancratinin C were detected in significant proportions [24]. The alkaloid 3-O-acetyl-narcissidine exhibited an antifeedant effect against the moth Spodoptera littoralis [25].

In addition, ultra-high-performance liquid chromatography coupled with high-resolution mass spectrometry (UHPLC-HRMS) was employed by our group to analyze ethanolic bulb extracts of *H. puniceum* due to their inhibitory properties against Dengue virus (DENV) and Zika virus (ZIKV). The MS/MS spectra of the active fractions against those viruses were consistent with compounds such as narcissidine acetate, narciclasine, lycorine, kalbreclasine, lycoramine E, lycoramine C, acetylnerbowdine, incartine, crisarnine, pseudolycorine, N-norlycoramine, and narciclasine-4-O-β-D-xylopyranoside (NXP) [26,27]. Nonetheless, as far as we currently know, there is no previous report in the literature on the anti-YFV activity of extracts and fractions from *H. puniceum* bulbs.

Therefore, the aim of this study was to explore the antiviral activities of *H. puniceum* bulb extract against a wild-type yellow fever virus (wt-YFV) utilizing bioassay-guided fractionation to identify new potential antiviral compounds using UHPLC-HRMS. Additionally, *in silico* predictions of pharmacokinetic and toxicity (ADMET) properties of the annotated compounds were evaluated to assess their potential for further *in vitro* and *in vivo* assessment. Indeed, computational analyses are fundamental in predicting the pharmacology of new drugs, providing a detailed understanding of essential properties such as absorption, distribution, metabolism, excretion, and toxicity (ADMET). These analyses not only assist in identifying potential issues with the studied compounds but also facilitate the prioritization of candidates and highlight areas for possible improvements in pharmaceutical development. Thus, they significantly contribute to the selection of candidate substances for *in vitro* and *in vivo* testing, aiding in the identification of those with the highest potential for success.

## 2. Results

### 2.1. Crude Extract Screening Against Wt-YFV

To evaluate the antiviral effect of the crude extract of *H. puniceum* against wt-YFV, Vero cells were infected with an m.o.i. of 0.1 and subsequently treated with the crude extract at 20 /mL. After 120 h, the MTT assay was performed and showed 58% protection of the treated cells compared to the untreated infected cells.

### 2.2. Bioassay-Guided Fractionation of H. puniceum Bulb Extract Against Wt-YFV

**Figure 1:**
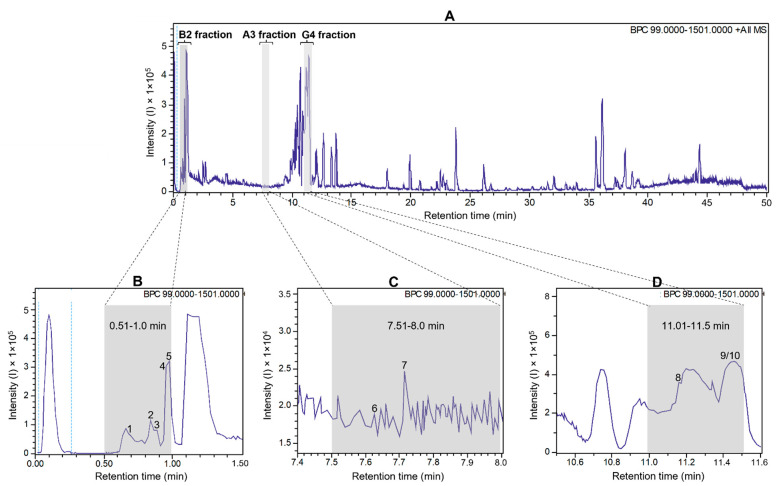
UHPLC-ESI-QTOF base peak chromatogram profiles of the ethanolic crude extract using a gradient elution of water and acetonitrile (ACN), both containing 0.1% v/v formic acid (5–100% ACN in 45 min; 100% ACN in 5 min) (**A**). Deconvoluted peaks for B2 fraction (**B**), A3 fraction (**C**), and G4 fraction (**D**) from *Hippeastrum puniceum*. Annotated compounds: see Table 1 for annotation and Figure 2 for chemical structures.

To detect antiviral compounds, the ethanolic bulb extract of *H. puniceum* was subjected to bioassay-guided fractionation using UHPLC-DAD as described in M&M. This approach yielded three fractions that exhibited activity against wt-YFV: fraction 02 from well B2, fraction 16 from well A3, and fraction 23 from well G4. These fractions demonstrated varying degrees of protection of cells infected with wild-type YFV, with fraction 02 showing 44% protection, fraction 16 showing 41% protection, and fraction 23 showing 51% protection. Importantly, none of these fractions displayed cytotoxic effects on Vero-CCL81 cells. These fractions are represented as gray bars in the base peak chromatogram of Figure 1, which was obtained in the first UHPLC run, as further described.

The parent ion ([M + H]^+^) with m/z 175.1190 showed a fragmentation pattern compatible with arginine, asparagine at m/z 133.0606, glutamic acid at m/z 148.0603, trigonelline at m/z 138.0547, cathinone at m/z 150.0911, tryptophan at m/z 205.0972, bulbisine at m/z 320.1490, tetrahydroharman-3-carboxylic acid at m/z 231.1126, 2,7-dimethoxyhomolycorine or 3-O-acetylnarcissidine at m/z 376.1755.

**Table 1:** Ions detected by UHPLC-ESI-MS/MS in the active fractions derived from the bioassay-guided fractionation of the ethanolic extract of *Hippeastrum puniceum* bulbs.

Fraction	Peak ID	RT (min)	Area (%)	Parent Ion [M + H]^+^ (m/z)	MS/MS Fragments (m/z, Relative Abundance -%)	Molecular Formula	Annotation
2 (well B2)-0.51–1.00 min	1	0.7	1.17	175.1190	175.1188 (100.0); 158.0922 (20.0); 130.0975 (9.7); 116.0698 (4.8)	C_6_H_14_N_4_O_2_	Arginine ^a,b^
	2	0.9	0.52	133.0606	133.0607 (100.0); 116.0348 (10.1); 132.1022 (7.8); 130.0505 (5.0)	C_4_H_8_N_2_O_3_	Asparagine ^a,b^
	3	0.9	0.41	148.0603	148.0603 (33.3); 146.1177 (37.5); 130.0498 (100.0); 99.4688 (1.1)	C_5_H_9_NO_4_	Glutamic acid ^b^
	4	1.0	1.10	138.0547	135.0676 (0.7); 136.0392(0.5); 136.0622 (0.5); 110.0899 (0.1)	C_7_H_7_NO_2_	Trigonelline ^a,b^
	5	1.0	1.06	150.0911	135.0676 (24.3); 134.0600 (9.1); 132.0807 (2.0); 119.0491 (3.2); 117.0 (1.7)	C_9_H_11_NO	Cathinone ^b^
16 (well A3)- 7.51–8.00 min	6	7.6	0.01	205.0972	188.0705 (100.0); 146.0598 (43.1); 132.0807 (10.8); 118.0650 (22.3)	C_11_H_12_N_2_O_2_	Tryptophan ^a,b^
	7	7.7	0.01	320.1490	302.1387 (12.1); 220.0750 (5.7); 147.0438 (34.6); 119.0489 (9.2);	C_17_H_21_NO_5_	Bulbisine ^b^
23 (well G4)- 11.01–11.50 min	8	11.1	10.94	231.1126	188.0705 (75.4); 158.0965 (100.0); 143.0723 (96.2); 130.0650 (52.8)	C_13_H_14_N_2_O_2_	Tetrahydroharman-3-carboxylic acid ^b,c^
	9/10	11.4	8.10	376.1755	376.1755 (100.0); 377.1789 (21.0); 165.0912 (16.3); 124.0754 (3.2);139.0543 (0.2)	C_20_H_25_NO_6_	3-O-acetyl narcissidine or 2,7-dimethoxyhomolycorine ^b,c^

^a^ Compounds annotated based on their predicted molecular formula, MS/MS fragments, and spectral comparison with public and in-house spectra libraries. ^b^ Known to occur in the Amaryllidaceae family. ^c^ Proposed by SIRIUS Software 5.5.7 (https://bio.informatik.uni-jena.de/sirius/). Accessed on 11 May 2025 [28].

**Figure 2:**
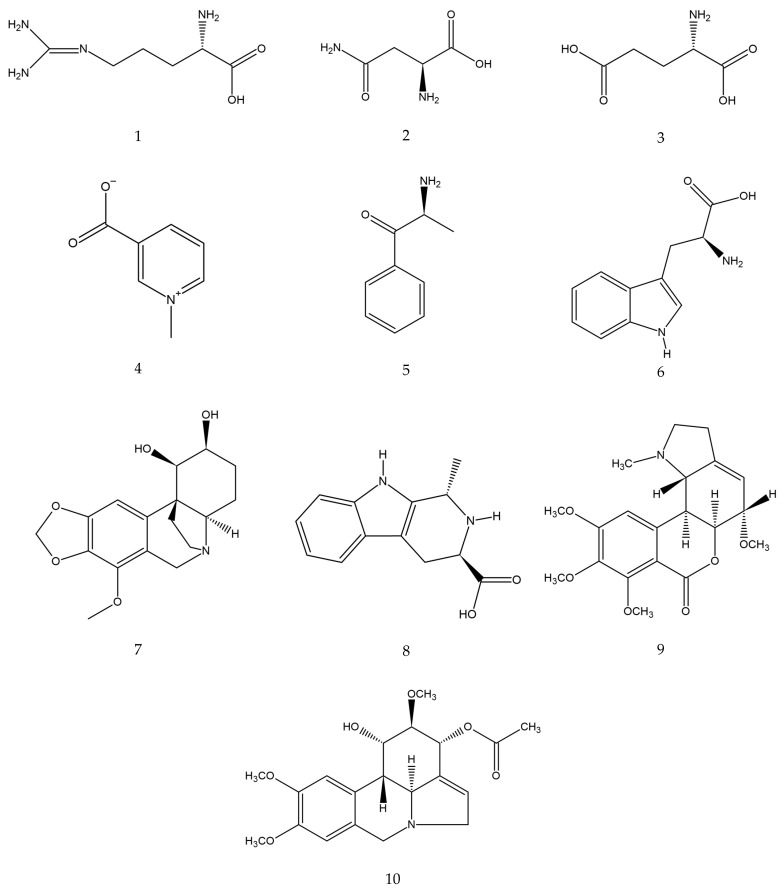
Estimated chemical structures of the annotated compounds: arginine (**1**), asparagine (**2**), glutamic acid (**3**), trigonelline (**4**), cathinone (**5**), tryptophan (**6**), bulbisine (**7**), tetrahydroharman-3-carboxylic acid (**8**), 2,7-dimethoxyhomolycorine (**9**), and 3-O-acetylnarcissidine (**10**).

### 2.3. In Silico ADMET Properties

In the present work, ADMET properties of the annotated alkaloids were computed using the pkCSM platform, aiming at predicting physicochemical attributes encompassing over 30 different ADMET parameters [29,30,31] (shown in Table 2) and considering Lipinski’s Rule of Five (RO5) [29]. The ADMET properties of the annotated amino acids were not shown since they are constituents of the primary metabolism.

The parameters established by the RO5 indicate that the molecular weight of a compound should not exceed 500 Da [29]. The calculated data in Table 2 shows that the alkaloids detected in this study fit within this parameter. When analyzing lipophilicity, the RO5 establishes a LogP value of ≤ 5, ideally between 1 and 4 [30,32]. The predicted values of this parameter for cathinone (LogP = 1.2165), tetrahydroharman-3-carboxylic acid (LogP = 1.8278), 3-O-acetylnarcissidine (LogP = 1.2328) and 2,7-dimethoxyhomolycorine (LogP = 1.9941) are within the described reference values.

According to the RO5, hydrogen bond acceptors and donors are also analyzed (the number of hydrogen bond acceptors should not exceed 10, and the number of donors should not exceed 5) [32]. Our ten compounds aligned with these parameters: trigonelline (acceptors: 2, donors: 0), cathinone (acceptors: 2, donors: 1), bulbisine (acceptors: 6, donors: 2), tetrahydroharman-3-carboxylic acid (acceptors: 2, donors: 3), 3-O-acetylnarcissidine (acceptors: 7, donors: 1) and 2,7-dimethoxyhomolycorine (acceptors: 7, donors: 0).

It is accepted that if an investigated compound does not meet two or more parameters analyzed in RO5, it is likely to have lower oral bioavailability, indicating the necessity to reassess its relevance in the context of drug development [33,34]. All the alkaloids analyzed in this study overcome this constraint, making them potential candidates for drug development against yellow fever.

Concerning the drug permeability in Caco2 cells, the values predicted for cathinone (log P_app_ = 1.237) and 2,7-dimethoxyhomolycorine (log P_app_ = 1.472) point to a potentially good oral absorption. The compounds tetrahydroharman-3-carboxylic acid and 3-O-acetylnarcissidine had predicted values close to the reference value (log P app > 0.90), with LogP app = 0.779; 0.832; and 0.705, respectively.

Considering the prediction of intestinal absorption, three compounds stand out for their high absorption rate: trigonelline, estimated to have 96.4% absorption rate, tetrahydroharman-3-carboxylic acid with 94.5%, and 2,7-dimethoxyhomolycorine with 97.7%. The compounds predicted to have intermediate intestinal absorption were cathinone (76.9%) and bulbisine (70.9%).

The calculated volume of distribution steady state (VDss), for which higher values indicate a broader distribution of the compound in tissues compared to plasma, shows that cathinone (log VDss = 0.465) has the highest predicted value, followed by 2,7-dimethoxyhomolycorine (VDss = 0.402) and bulbisine (VDss = 0.401). This volume represents the theoretical volume required for the total dose of a drug to be evenly distributed throughout the body, reaching a concentration equivalent to that found in plasma [35].

The capacity of molecules to cross the blood–brain barrier (BBB) and enter the central nervous system (CNS) is crucial, and depending on the therapeutic objective, the drug’s ability to reach the brain might not be desirable; thus, a negative result for this parameter is preferred [32]. Among the alkaloids investigated, tetrahydroharman-3-carboxylic acid was predicted to have the highest ability to cross the blood–brain barrier (log BB = 0.225).

The cytochrome P450 (CYP450) family involves enzymes responsible for drug metabolism, performing biotransformation of a myriad of compounds. Predominantly located in the liver, CYP450 enzymes play a crucial role in detoxifying the body by oxidizing xenobiotics to facilitate their excretion [35,36]. The CYP450 system consists of isoenzymes grouped and classified into subfamilies based on their amino acid sequence similarities. The CYP1, CYP2, and CYP3 families have been described as the most significant in drug biotransformation. Among these families, the isoenzymes 1A2, 2C9, 2C19, 2D6, and 3A3/4 are the most significantly involved in drug metabolism [37].

Tetrahydroharman-3-carboxylic acid was identified as a potential substrate for the CYP2D6 isoenzyme, which is involved in the metabolism of fatty acids, steroids, and retinoids [32]. CYP3A4 is involved in the metabolism of sterols, steroid hormones, retinoids, and fatty acids [32]. Among the alkaloids, bulbisine, 3-O-acetylnarcissidine, and 2,7-dimethoxyhomolycorine emerged as putative substrates and may be metabolized by this pathway. Concerning CYP1A2, which is involved in the metabolization of endogenous substrates such as fatty acids, steroid hormones, and vitamins [32], cathinone was predicted to act as an inhibitor. Finally, none of the analyzed molecules showed potential as inhibitors for CYP2C9, CYP2D6 and CYP3A4 enzymes.

The predicted excretion profiles showed that bulbisine would exhibit higher clearance capacity (logCL = 1.137), consistent with its predicted metabolization, which is foreseen to be a substrate of CYP3A4 and excreted after P450-mediation.

The decreasing order of clearance of the other alkaloids was bulbisine (logCL = 1.137), 3-O-acetylnarcissidine (logCL = 0.902), cathinone (logCL = 0.811), tetrahydroharman-3-carboxylic acid (logCL = 0.694), 2,7-dimethoxyhomolycorine (logCL = 0.636), trigonelline (logCL = 0.378).

None of the proposed alkaloids were predicted to induce genetic mutations (AMES toxicity) or cause skin sensitization. However, 3-O-acetylnarcissidine and bulbisine were identified as potentially hepatotoxic. Regarding the values predicted for the maximum recommended therapeutic dose (MRTD), 3-O-acetylnarcissidine showed the highest [−0.558log (mg/kg/day)], followed by the compound 2,7-dimethoxyhomolycorine [−0.099log (mg/kg/day)].

### 2.4. CC_50_ and EC_50_ Values of Annotated Compounds Obtained from the Bioassay-Guided Fractionation of Hippeastrum Puniceum Bulb Extract

Of the ten compounds identified in the bioactive fractions, six were obtained from commercial sources: asparagine, arginine, glutamic acid, tryptophan, trigonelline, and bulbisine. These compounds were evaluated for cytotoxicity and antiviral activity against wt-YFV in Vero-CCL81 cells. In addition to these compounds, the crude extract was also re-evaluated, presenting CC_50_ = 5.2 µg/mL and EC_50_ = 4.0 µg/mL. Interferon-alpha served as an internal control (Figure S1). Asparagine, arginine, and trigonelline exhibited low toxicity, with CC_50_ values close to 1000 µM, suggesting a favorable cellular safety profile. In contrast, bulbisine, tryptophan, and glutamic acid showed significant cytotoxicity, with CC_50_ values of 123 µM, 578 µM, and 613 µM, respectively (Figure S2). However, none of the tested compounds demonstrated antiviral activity against wt-YFV under the evaluated conditions.

## 3. Discussion

Aiming at disclosing new potential antivirals against wt-YFV, a bioassay based on the replication of this virus on Vero cells was used to guide the fractionation of the crude extract of *H. puniceum* bulb using ultra-high performance liquid chromatography coupled to high-resolution mass spectrometry. With the help of computational analysis using the pkCSM platform, predictions of the pharmacological potential of the bioactive compounds were generated.

Previous work with this plant species in our group showed the activity of *H. puniceum* bulb extract against Zika virus and Dengue virus, where the alkaloids lycorine, pseudolycorine, pancracine, nangustine, pretazettine, narcissidine acetate, marciclasine, kalbreclasine, lycoranine E, lycoranine C, acetylnerbowdine, incartine, crisarnine, N-norlycoramine, and narciclasine-4-O-β-D-xylopyranoside were detected in the active fractions [26]. Here, we used the bioassay with wt-YFV to identify fractions capable of controlling viral replication, as the crude extract showed 58% protection in the antiviral assays. Among the eighty fractions collected, three were considered active.

Based on the analysis of the high-resolution mass spectrometric data of these three fractions, we found that trigonelline and cathinone were present in the most polar fraction (RT 0.5–1 min), together with arginine, asparagine, and glutamic acid. In the second active fraction (RT 7.5–8 min), we were able to propose the presence of bulbisine and tryptophan. In the last fraction, with RT 11–11.5 min, the presence of the alkaloids 3-O-acetylnarcissidine, tetrahydroharman-3-carboxylic acid and 2,7-dimethoxyhomolychorin was proposed.

The presence of free amino acids, such as glutamine, arginine, threonine, asparagine, and alanine, was previously described in protoplasts of *Hippeastrum* [38]. Amino acids play a crucial role as gene expression regulators. Physiological concentrations of amino acids and their metabolites, such as nitric oxide, polyamines, glutathione, taurine, thyroid hormones, and serotonin, are necessary for their proper functions [39]. Indeed, antiviral properties of some amino acids have been reported.

Glutamic acid, a precursor of glutamine, plays crucial metabolic and signaling roles in the human body, including neurotransmission, energy metabolism, and regulation of acid-base balance [40,41]. A polymer, poly-γ-glutamic acid (γ-PGA), induced innate immune responses through the TLR4-MD2 complex, resulting in an antiviral state effective against SARS coronavirus and hepatitis C virus [42]. Further studies are needed to investigate the antiviral activity of this amino acid against YFV.

Asparagine and aspartate metabolism has been suggested to be important to fine-tune macrophage-mediated inflammation; however, there are no reports of their antiviral properties [43]. Their function might be particularly significant in the context of YF pathogenesis since YFV infects lymphoid cells and macrophages, where it carries out its replicative cycle [44]. Hence, targeting cellular signaling pathways potentially influenced by asparagine may hold promise as an important therapeutic strategy for YF management.

Tryptophan, an essential amino acid, was detected in extracts of *Narcissus tazetta* (Amaryllidaceae) using an ultra-performance liquid chromatography-diode array detection method [45]. Tryptophan and its metabolites play key roles in various physiological processes; however, its antiviral action alone or in combination with other compounds still requires investigation.

A number of alkaloids have significant antiviral properties against various infectious viruses, inhibiting important steps of viral replication, indicating that they could serve as effective and safe antiviral medications if further pursued in medicinal and pharmacological investigations [46].

Trigonelline is an alkaloid found in plants of the Amaryllidaceae family, such as *Narcissus pseudonarcissus* L. [47] and in *Areca vestiaria* (Arecaceae) [48]. More and colleagues (2022) also identified this alkaloid in plant extracts with antiviral properties against Rift Valley fever virus (RVFV) obtained from *Adansonia digitata* (Malvacea), *Elephantorrhiza elephantina* and *Sutherlandia frutescens* (Fabacea) using proton nuclear magnetic resonance spectroscopy (^1^H NMR) coupled with multivariate data analysis (MVDA) [49]. Trigonelline significantly inhibited the survival of herpes simplex virus (type 1) [50]. Our *in silico* calculations predicted that trigonelline could have low mutagenic activity, high intestinal absorption rate and intermediate ability to penetrate the CNS. Together, these data encourage further in vitro and in vivo investigations of this alkaloid against YFV.

Cathinone is described here for the first time in the Amaryllidaceae family. This compound is found in *Catha edulis* leaves and possesses psychoactive effects like amphetamines, earning it the nickname “natural amphetamine” due to its structural and pharmacological resemblance [51]. However, current studies have not reported the potential use of cathinones as antiviral agents. There is a scarcity of studies exploring the potential use of cathinones as antiviral agents, however lead screening for HIV-1 integrase inhibition by simulating molecular docking and molecular dynamics selected cathinone as a putative antiviral against this virus [52]. Cathinone was predicted to have good oral absorption, moderate intestinal and cutaneous absorption. Additionally, it showed the highest prediction for a steady-state volume of distribution, indicating widespread distribution in body tissues and compartments, including the central nervous system.

Bulbisine, also named bowdensine, is found in bulbs of *Hippeastrum littoralis* [53] and *Nerine sarniensis,* another Amaryllidaceae [54]. So far, no biological and antiviral properties of this alkaloid have been reported. ADMET prediction indicates that bulbisine is unlikely to penetrate the central nervous system, has intermediate intestinal absorption, and is potentially hepatotoxic. This prediction is consistent with our *in vitro* results, in which the compound exhibited significant cytotoxicity in Vero cells, with a CC_50_ value of 123 µM. Yet, bulbisine may be structurally modified to enhance its antiviral activity and reduce cytotoxicity, as well as to optimize other pharmacokinetic properties.

The presence of tetrahydroharman-3-carboxylic acid, a derivative of tetrahydro-β-carboline, is reported here for the first time in an Amaryllidaceae species. Although there is no information on the antimicrobial activity of tetrahydroharman-3-carboxylic acid. Its precursor, tetrahydro-β-carboline, exhibits well-documented antioxidant activities [55]. The computational analysis for tetrahydroharman-3-carboxylic acid predicted a high likelihood of being absorbed in the intestine, easily crossing the blood–brain barrier, nor capable of inducing genetic mutations.

The presence of 3-O-acetylnarcissidine in *H. puniceum* was previously described by Santana and colleagues (2008) [25]. However, we could not find any report related to its biological activity, making it a good candidate for additional investigations to assess its efficacy against YFV. In Silico prediction can give us a glimpse of the physicochemical properties of 3-O-acetylnarcissidine. ADMET prediction indicated a good oral absorption and moderate intestinal and cutaneous absorption, with little likelihood of penetrating the CNS. However, it was predicted to have the lowest MRTD among the alkaloids analyzed, besides its potential hepatotoxicity.

2,7-Dimethoxyhomolycorine is a derivative of the alkaloid lycorine. Previous studies conducted by our group indicated that lycorine exhibits antiviral activity against dengue virus type 2 (DENV-2) and Zika virus (ZIKV) [27]. These findings are consistent with previous studies reporting the in vivo antiviral activity of lycorine against ZIKV in AG6 mice, which resulted in a lower viral load in the blood and reduced mortality [56]. Lycorine has also demonstrated broad-spectrum antiviral activity against various other viruses, including poliovirus [57], herpes simplex virus type 1 [58], SARS-CoV [59], and West Nile virus [60]. These data encourage us to investigate the antiviral activity of 2,7-dimethoxyhomolycorine against wt-YFV.

Although tests with the compounds asparagine, arginine, glutamic acid, tryptophan, trigonelline, and bulbisine did not reveal significant antiviral activity, the protective effect observed in the bioactive fractions suggests a synergistic interaction among multiple compounds. These findings highlight the potential of *H. puniceum* bulb as a source of novel antiviral agents against wt-YFV and underscore the importance of investigating the combinatorial effects of its constituents. Future studies, including structural optimization of the promising alkaloids, are necessary to fully elucidate their mechanisms of action and therapeutic potential.

## 4. Materials and Methods

### 4.1. Reagents and Solvents

All reagents used in this study were of analytical grade. The absolute ethanol used for extract production was purchased from LS Chemicals, Ribeirão Preto, Brazil. Dimethyl sulfoxide (DMSO) and dimethyl-2-thiazolyl tetrazolium bromide (MTT) were purchased from Sigma-Aldrich, Saint-Quentin-Fallavier, France, and formic acid was purchased from Fluka, Muskegon, MI, USA. Acetonitrile and 2-propanol were purchased from Merck, Supelco, Darmstadt, Germany. The water was purified using the Milli-Q IQ7000 ultrapure water system, Merck Millipore, Burlington, MA, USA.

Some annotated substances were obtained from commercial suppliers for biological evaluation, namely: L-arginine monohydrochloride (Sigma-Aldrich, Saint Louis, MO, USA, #A5131); L-asparagine monohydrate (#A4284); L-glutamic Sigma-Aldrich, Saint Louis, MO, USA, #G1251); trigonelline hydrochloride (Sigma-Aldrich, Saint Louis, MO, USA, #61407); bulbisine (BOC Sciences, Shirley, NY, USA, #101219-55-0) and DL-tryptophan (Sigma-Aldrich, Saint Louis, MO, USA,#16269-8).

### 4.2. Cells, Wild-Type YFV, and Interferon Alfa-2a

C6/36 cells derived from *Aedes albopictus* mosquito larvae, obtained from the Rio de Janeiro Cell Bank (BCRJ, Rio de Janeiro, Brazil #0343), were used for viral multiplication. These cells were cultured in Leibovitz’s L-15 medium (41300039, Thermo Fisher Sci, Waltham, MA, USA), supplemented with 2% fetal bovine serum (FBS) (Gibco, São Paulo, Brazil) and 100 U/mL of penicillin/streptomycin (1514012, Thermo Fisher Sci, Waltham, MA, USA). Vero CCL-81 cells, derived from the kidney of a normal adult African green monkey (*Cercopithecus aethiops*), were purchased from BCRJ (Rio de Janeiro, Brazil #0245).

Vero cells were cultured in Dulbecco’s Modified Eagle Medium with low glucose content (DMEM) (#31600034, Gibco, São Paulo, Brazil), supplemented with 2% FBS and 100 U/mL of penicillin/streptomycin, at 37 °C in a 5% CO_2_ atmosphere incubator (Thermo Scientific, Waltham, MA, USA). These cells were used for viral titration and for cytotoxicity and antiviral activity assays. The wild-type yellow fever virus (wt-YFV) used in this study was isolated from a hospitalized patient during the yellow fever outbreak in Minas Gerais State, Brazil, in 2017. A working stock of low passage of wt-YFV in C6/36 cells was stored at −80 °C and titrated by plaque formation assay in Vero cells as described [61].

Human recombinant interferon alfa-2a (IFN-α-2a) from (INREC, Uruguay) was used as a positive control in all antiviral assays.

### 4.3. Plant Material

Bulbs of *Hippeastrum puniceum* (Lam.) Kuntze were collected by Dr. Carlos Alberto Ferreira Junior at Fundação Zoobotânica, located in the municipality of Belo Horizonte, Minas Gerais State, Brazil, in April 2022. Carlos Alberto Ferreira Junior was also responsible for the identification of plant specimens under the voucher code BHZB 12069. The access to the genetic heritage of the plant has been registered in the National System of Genetic Heritage Management (SISGEN) under the number AF030EA.

### 4.4. Ethanolic Extract Preparation

Briefly, 300 g bulbs of *H. puniceum* were cut into small pieces, transferred to a 1000 mL flask, which was then filled with absolute ethanol. The plant material was then left in contact at room temperature in the dark for one week. The macerate was filtered, and the solvent was removed at 40 °C under vacuum, affording 16.7 g (5.6%) of crude extract. A stock solution of the crude extract at 20 mg/mL was prepared using 90% DMSO and stored at −20 °C until being used in the experiments.

### 4.5. Bioassay-Guided Chromatographic Fractionation

A Nexera ultra-high-performance liquid chromatograph equipped with a Shim-Pack XR-ODS-III column (C18, 2.2 μm, 2.0 × 150 mm, Shimadzu, Kyoto, Japan) held at 40 °C was used for the fractionation of the *H. puniceum* extracts. The column effluent was directed to a diode-array detector followed by a MaXis ETD high-resolution ESI-QTOF mass spectrometer (Bruker, Bremen, Germany). This setup was controlled by the Compass 1.7 software package (Bruker, Bremen, Germany). The mobile phase, composed of a mixture in different proportions of A (0.1% formic acid) and B (acetonitrile with 0.1% formic acid), was pumped at a flow rate of 400 μL/min. The chromatography started with 5% B for 5 min, then reached 100% B in 45 min and held at this proportion for 5 min. The mass spectra were acquired in positive mode at a spectra rate of 5 Hz. Ion-source parameters were set to 500 V endplate offset, 4500 V capillary voltage, 3.0 bar nebulizer pressure, 8 L/min, and 200 °C dry gas flow and temperature, respectively. Data-dependent MS/MS was run using a collision energy range between 15 and 60 eV. Ion cooler settings were optimized for an m/z 100–1500 range using a calibrant solution of 1 mM sodium formate in 50% 2-propanol. Mass calibration was achieved by initial ion-source infusion of 20 μL of sodium formate calibrant solution and post-acquisition recalibration of the raw data. Compound detection was performed by chromatographic peak dissection with subsequent formula prediction using Bruker Compass DataAnalysis 4.2 software (Bruker, Bremen, Germany). Putative identification (annotation) was based on the comparison of compound fragment spectra (MS2) with in-house and public spectral libraries, and with standard compounds [62].

The sample was diluted in DMSO/acetonitrile to a final concentration of 5 mg/mL. For spectral analysis, 3 μL (15 μg) were fractionated using the above conditions, and the UV and HRMS, and MS/MS data were recorded. To collect fractions for the bioassays while keeping identical retention times with the previous run, 10 μL (50 μg) of the extract were used, and the column effluent was directed to an automatic fraction collector (Advantec, Dublin, CA, USA) without passing through the mass detector. Eighty 200 μL fractions were collected in 96-well polypropylene microtiter plates. These plates were placed in a vacuum centrifuge at 40 °C (Thermo Savant, Asheville, NC, USA) to remove all solvents before being used in the bioassays. The bioactive fractions were chemically characterized by HRMS for putative compound identification.

### 4.6. Cytotoxicity Assay and Wt-YFV Replication Inhibition Assay

The crude extract of *H. puniceum* and all chromatographic fractions were analyzed for their cytotoxic and anti-wt-YFV activities in Vero-CCL81 cells.

For cytotoxicity assays, cells were seeded in 96-well plates at a density of 1 × 10^4^ cells per well in complete DMEM medium and incubated at 37 °C in a 5% CO_2_ atmosphere for 24 h until reaching approximately 80% confluence. After, cells were treated with the crude extract at a final concentration of 20 μg/mL in DMEM medium supplemented with 2% fetal bovine serum (FBS). The concentration of 20 μg/mL for the crude extract was determined according to earlier bioprospecting results reported previously [27]. The plates were then incubated at 37 °C in a 5% CO_2_ atmosphere for five days.

For the wt-YFV replication inhibition assay, a 100 µL volume of viral suspension with an m.o.i. of 0.1 was added to the cell monolayers at 80% confluence. This m.o.i. was defined based on prior standardizations conducted by our research group and supported by previous studies in the literature [63,64,65]. Viral adsorption was performed for 1 h, followed by treatment of the samples at the same concentration used in the cytotoxicity assays. The final volume in each well was adjusted to 200 µL with DMEM, and the plates were incubated at 37 °C and 5% CO_2_ atmosphere.

Cytotoxic and cytopathic effects were observed daily by optical microscopy. Each sample was evaluated in duplicate. Control sets of untreated infected and untreated non-infected cells with and without DMSO addition were included. IFN-α-2a, at a final concentration of 600 U/mL, was used as a positive antiviral control in all antiviral assays. Cytotoxicity and antiviral assays were revealed by the MTT colorimetric method [66]. Briefly, the supernatant was removed and replaced with 30 µL per well of an MTT solution (2 mg/mL) dissolved in phosphate-buffered saline. The MTT-treated cells were then incubated for 90 min at 37 °C. Subsequently, 130 µL of DMSO was added to each well, and the plates were homogenized for 5 min at 500 rpm. Absorbance values of each reaction were measured using an ELISA reader (Spectra Max, Molecular Devices, San Jose, CA, USA) at λ 540 nm. After five days, the reduction in the viral cytopathic effect (CPE) and the cytotoxic effect were assessed. The extract or fractions were considered active if they showed at least 40% protection.

### 4.7. In Silico ADMET Prediction

The pharmacokinetic parameters, including absorption, distribution, metabolism, excretion, and toxicity (ADMET), of the compounds detected in the active fractions against wt-YFV were predicted using the pkCSM platform (https://biosig.lab.uq.edu.au/pkcsm/prediction) (accessed on 27 March 2024) [35]. pkCSM is a free-to-use, user-friendly machine-learning platform that leverages graph-based signatures to build predictive models via supervised learning for over 30 ADMET endpoints [67]. The chemical structures of the annotated compounds were provided to the web-based platform in their canonical representation using the Simplified Molecular Input Line Entry System (SMILES).

### 4.8. Determination of CC_50_ and EC_50_ of Annotated Compounds from the Bioassay-Guided Fractionation of Hippeastrum Puniceum Bulb Extract

Based on the annotation of the compounds present in the active fractions against wt-YFV, the CC_50_ and EC_50_ of trigonelline, bulbisine, arginine, asparagine, glutamic acid, and tryptophan were determined using Vero-CCL81 cells.

All compounds, except bulbisine, were tested at 12 different concentrations, with a maximum concentration of 1000 µM. For bulbisine, the highest concentration tested was 250 µM. Initially, serial dilutions of the test substances were prepared in sterile plates (ranging from 1000 to 0.5 µM or from 250 to 0.125 µM, depending on the compound). These dilutions were then transferred to plates containing previously cultured monolayers of 1.0 × 10^4^ Vero cells, either infected or uninfected with wt-YFV (m.o.i. 0.1).

The plates were incubated for five days at 37 °C in a controlled atmosphere with 5% CO_2_. At the end of the incubation period, antiviral activity and cytotoxicity were evaluated by microscopic inspection of the cytopathic effect using the cross-scoring method, and by the MTT colorimetric assay, as described in Section 4.6. The data obtained were statistically analyzed using GraphPad Prism software, version 8.0 (GraphPad Software, Boston, USA). Dose–response curves were fitted using nonlinear regression to determine the CC_50_ and the EC_50_.

### 4.9. Statistical Analyses

Statistical analyses were performed using the GraphPad Prism 8 software. The Shapiro–Wilk normality test was previously applied to all data. The Two-way ANOVA test followed by Sidak multiple comparisons were used to analyze the parametric data. For non-parametric data, the Kruskal–Wallis test was applied, followed by Dunn’s multiple comparisons test. Differences were considered significant when the *p*-value was less than or equal to 0.05.

## 5. Conclusions

In conclusion, the integration of bioassay-guided fractionation with UHPLC-HRMS/MS analysis of a *Hippeastrum puniceum* bulb extract exhibiting significant antiviral activity against wild-type yellow fever virus (wt-YFV) enabled the annotation of six alkaloids and four amino acids. The results suggest that the antiviral activity of the extract may arise from synergistic interactions among multiple constituents, since the individual compounds did not exhibit notable anti-YFV effects in vitro. Further studies are required to elucidate these interactions. In silico ADMET analyses indicated favorable pharmacokinetic and toxicological properties for the identified compounds, supporting their potential as lead candidates for antiviral drug development. Overall, these findings highlight the effectiveness of integrating bioassay-guided fractionation with computational approaches as a strategic framework for the discovery of natural products with therapeutic potential and for advancing the development of novel antiviral agents. Indeed, this work expands current knowledge of the chemical diversity and pharmacological potential of *H. puniceum* and reinforces the relevance of natural products as valuable sources for the discovery of new therapeutic agents targeting yellow fever, a persistent and reemerging viral disease.

## Figures and Tables

**Table 2:** Predicted pharmacokinetic and toxicity (ADMET) properties of the alkaloids present the bioactive fractions against wt-YFV using the pkCSM platform.

PARAMETERS	COMPOUNDS						INDICATORS
	Trigonelline	Cathinone	Bulbisine	Tetrahydroharman-3-carboxylic Acid	3-O-acetyl-narcissidine	2,7-dimethoxy-homolycorine	
MOL_WEIGHT	137,138	149,193	319,357	230,267	375,421	375,421	
LOGP	−1.1254	1.2165	0.7652	1.8278	1.2328	1.9941	Lipinski’s RO5: <5 ideally between 1–4
ROTATABLE_BONDS	1	2	1	1	4	4	
ACCEPTORS	2	2	6	2	7	7	
DONORS	0	1	2	3	1	0	
SURFACE_AREA	58,547	66,028	134,111	98,647	158,003	158,323	
Water solubility	−1.931	−0.795	−1.859	−2.435	−2.948	−3.146	The predicted water solubility of a compound is given as the logarithm of the molar concentration (log mol/L).
Caco2 permeability	1.124	1.237	−0.138	0.832	0.705	1.472	High Caco2 permeability would translate into predicted values >0.90
Intestinal absorption (human)	96.44	76.876	70.972	94.534	64.054	97.738	Poorly absorbed: <30%
Skin Permeability	−2.736	−2.278	−3.236	−2.735	−3.175	−2.887	Low skin permeability if it has a logKp > −2.5.
P-glycoprotein substrate	Yes	No	Yes	Yes	Yes	No	Yes or No
P-glycoprotein I inhibitor	No	No	No	No	No	No	Yes or No
P-glycoprotein II inhibitor	No	No	No	No	No	No	Yes or No
VDss (human)	−0.758	0.465	0.401	−0.237	0.481	0.402	Low if below 0.71 L/kg (log VDss < −0.15) and high if above 2.81 L/kg (log VDss > 0.45).
Fraction unbound (human)	0.857	0.469	0.436	0.481	0.413	0.411	For a given compound, the predicted fraction that would be unbound in plasma will be calculated.
BBB permeability	−0.234	−0.133	−0.716	0.225	−0.538	−0.396	Readily cross BBB > 0.3; Poorly distributed in brain < −1
CNS permeability	−2.739	−1.768	−3.429	−3.254	−3.243	−2.969	Can penetrate, Log PS > −2 Cannot penetrate, Log PS < −3
CYP2D6 substrate	No	No	No	Yes	No	No	Yes or No
CYP3A4 substrate	No	No	Yes	No	Yes	Yes	Yes or No
CYP1A2 inhibitor	No	Yes	No	No	No	No	Yes or No
CYP2C19 inhibitor	No	No	No	No	No	No	Yes or No
CYP2C9 inhibitor	No	No	No	No	No	No	Yes or No
CYP2D6 inhibitor	No	No	No	No	No	No	Yes or No
CYP3A4 inhibitor	No	No	No	No	No	No	Yes or No
Total Clearance	0.378	0.811	1.137	0.694	0.902	0.636	-
Renal OCT2 substrate	No	No	No	No	No	No	Yes or No
AMES toxicity	No	No	No	No	No	No	Yes or No
Max. tolerated dose (human)	0.743	0.779	0.025	0.323	−0.558	−0.099	High is greater than 0.477
hERG I inhibitor	No	No	No	No	No	No	Yes or No
hERG II inhibitor	No	No	No	No	No	No	Yes or No
Oral Rat Acute Toxicity (LD50)	1.878	2.131	3.124	2.412	3.109	2.739	The LD50 is the amount of a compound given all at once that causes the death of 50% of a group of test animals.
Oral Rat Chronic Toxicity (LOAEL)	0.454	1.542	2.614	1.115	1.610	2.689	The LOAEL results need to be interpreted relative to the bioactive concentration and treatment lengths required.
Hepatotoxicity	No	No	Yes	No	Yes	Yes	Yes or No
Skin Sensitisation	No	No	No	No	No	No	Yes or No

Legend: LogP: logarithm of the partition coefficient; Caco2: Human colorectal adenocarcinoma cells;VDss: volume of distribution at steady state; BBB: Blood–Brain Barrier; CNS: Central nervous system; CY: Cytochrome P450 substrates; OCT2: Renal organic cation transporter 2; AMES: The name of the test used in mutagenicity assessment. hERG: Human ether-go-go gene; LD50: LethalDose for 50%; LOAEL: Lowest observed adverse effect level.

## Data Availability

The data supporting the reported results originates from the master’s thesis of the author E.F.S. However, the data is not yet publicly available due to privacy restrictions. The thesis abstract can be accessed at: https://www.arca.fiocruz.br/handle/icict/67216 (accessed on 14 September 2025).

## Supplementary Materials

The following supporting information can be downloaded at: https://www.mdpi.com/article/10.3390/molecules30214149/s1, Figure S1: Determination of CC_50_ and EC_50_ Values of *Hippeastrum puniceum* Bulb Extract; Figure S2: Determination of CC_50_ and EC_50_ of Annotated Compounds from the Bioassay-Guided Fractionation of *Hippeastrum puniceum* Bulb Extract.

## Abbreviations

The following abbreviations are used in this manuscript:

ACN	Acetonitrile
ADMET	Absorption, distribution, metabolism, excretion, and toxicity
AMES	The name of the test used in mutagenicity assessment
ATCC	American Type Culture Collection
BBB	Blood–brain barrier
BCRJ	Rio de Janeiro Cell Bank
Caco2	Human colorectal adenocarcinoma cells
CC_50_	50% Cytotoxic Concentration
CL	Clearance capacity
CNS	Central nervous system
CPE	Cytopathic effect
CYP450	Cytochrome P450
DENV	Dengue Virus
DMEM	Dulbecco’s Modified Eagle Medium
DMSO	Dimethyl Sulfoxide
EC_50_	50% Effective Concentration
FBS	Fetal bovine serum
GC-MS	Gas chromatography-mass spectrometry
hERG	Human ether-go-go gene
HIV	Human Immunodeficiency Virus
ICTV	International Committee on Taxonomy of Virus
IFN-α-2a	Human recombinant interferon alfa-2a
LD50	Lethal Dose for 50%.
LOAEL	Lowest observed adverse effect level
LogP	Logarithm of the partition coefficient
M.O.I	Multiplicity of infection
MRTD	Maximum recommended therapeutic dose
MTT	(3-(4,5-Dimetil-2-tiazolyl)-2,5-Difeniltetrazólio)
MVDA	Multivariate data analysis
NPX	Narciclasine-4-O-β-D-xylopyranoside
NS	Non-structural
OCT2	Renal organic cation transporter 2
RO5	Lipinski’s Rule of Five
RT	Retention time
RVFV	Rift Valley Fever Virus
SARS	Severe acute respiratory syndrome
SISGEN	National System of Genetic Heritage Management
SMILES	Simplified Molecular Input Line Entry System
TMEM41B	Transmembrane Protein 41B
TRIM56	Tripartite Motif Containing 56
UHPLC	Ultra-high-performance liquid chromatograph
VDss	Volume of distribution at steady state
wt-YFV	Wild-type yellow fever virus
YF	Yellow fever
YFV	Yellow Fever Virus
ZIKV	Zika Virus
γ-PGA	polymer poly-γ-glutamic acid
