# Supplementary material for: Potential Antiviral Compounds from Hippeastrum puniceum Bulb Against Yellow Fever Virus: Bioassay-Guided Fractionation and In Silico Pharmacokinetic Analysis
Source: Molecules. 2025 Oct 22;30(21):4149. doi: 10.3390/molecules30214149 (PMC12608337; doi:10.3390/molecules30214149)
Supplement: Supplementary file 1 [file molecules-30-04149-s001.zip › molecules-3904498-supplementary.pdf]

Figure S1: Determination of CC_{50} and EC_{50} Values of *Hippeastrum puniceum* Bulb Extract

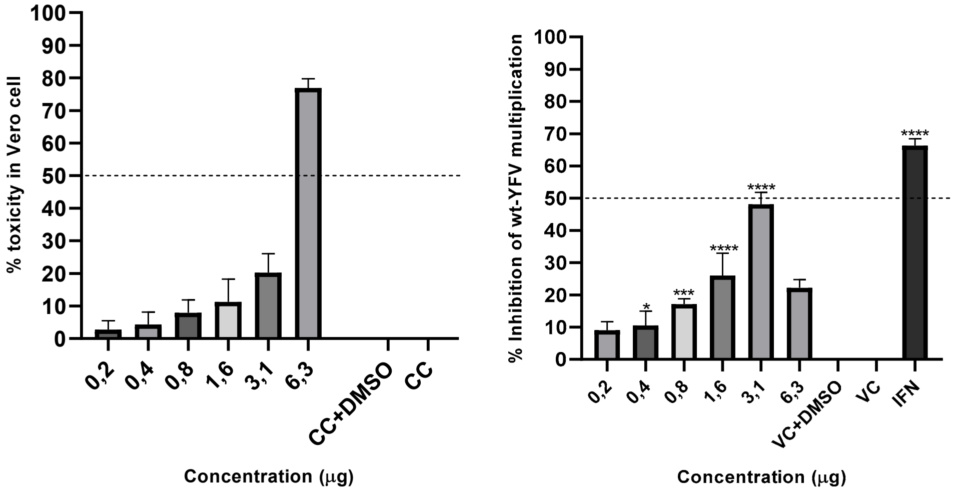

Percentage of toxicity and wt-YFV inhibition after treatment of Vero cells with different concentrations of *Hippeastrum puniceum* bulb extract. DMSO was used as the vehicle control and IFN as the positive control. The dashed line indicates the point corresponding to 50% toxicity or viral inhibition. CC+DMSO: Cell control with DMSO; CC: Cell control; VC+DMSO: Viral control with DMSO; VC: Viral control; IFN: Interferon.

Figure S2: Determination of CC_{50} and EC_{50} of Annotated Compounds from the Bioassay-Guided Fractionation of *Hippeastrum puniceum* Bulb Extract

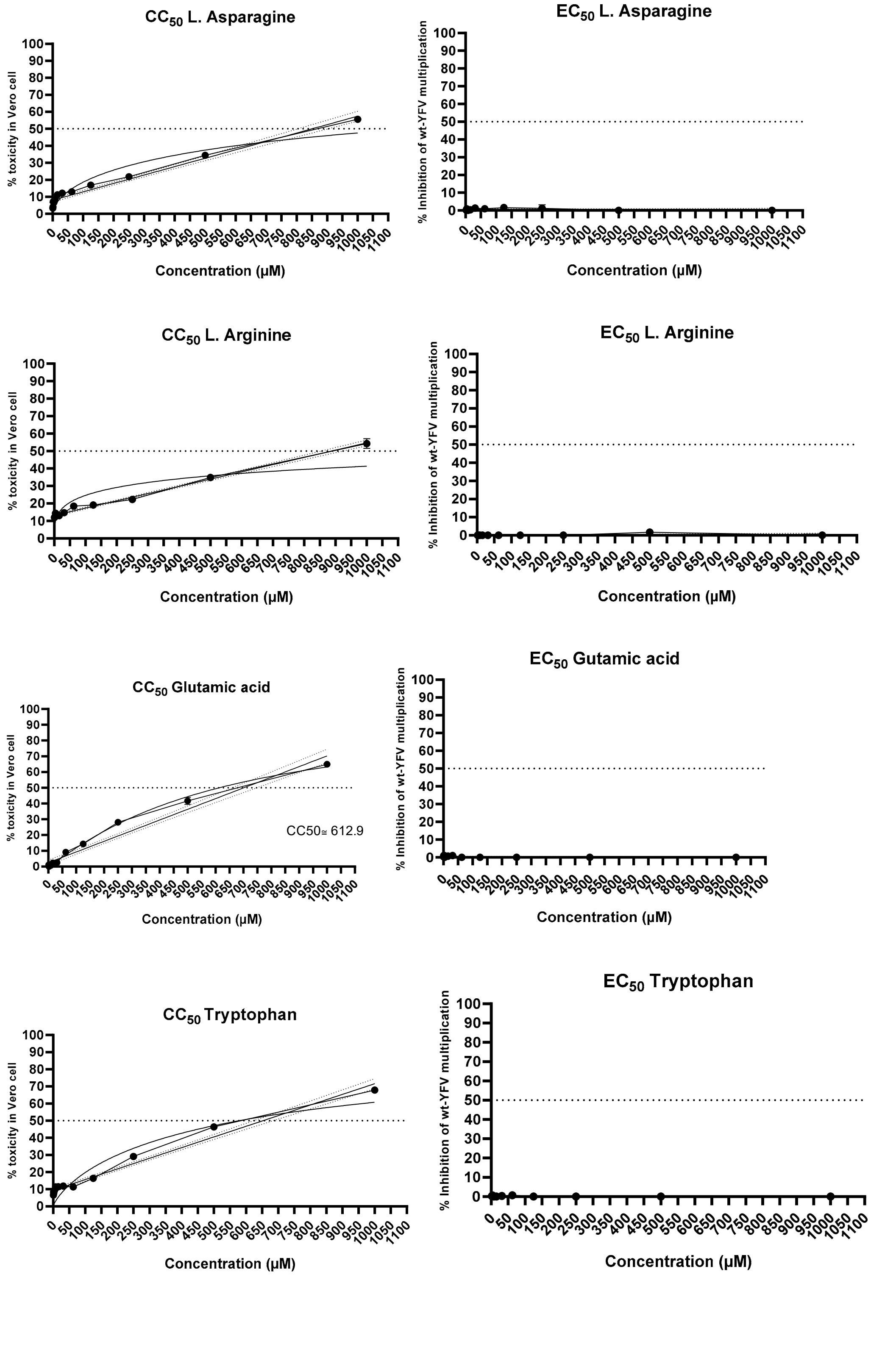

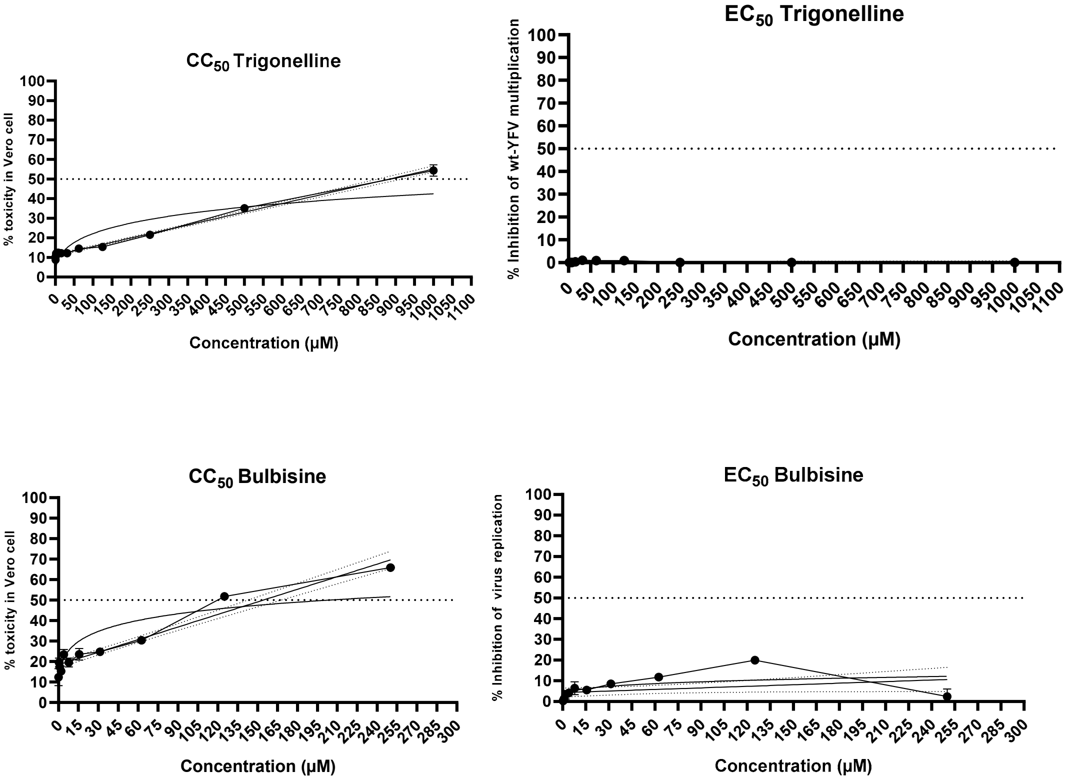

Determination of CC_{50} and EC_{50} values of annotated compounds obtained from the bioassay-guided fractionation of *Hippeastrum puniceum* bulb extract. Vero cells were treated with increasing concentrations of the purified compounds to assess cytotoxicity (CC_{50}) and antiviral activity against wt-YFV (EC_{50}). DMSO was used as vehicle control. The dashed lines indicate the points corresponding to 50% cytotoxicity or 50% viral inhibition.
